# An Efficient Measure of Sexual Interest in Children: The Revised Screening Scale for Pedophilic Interests (SSPI-2)

**DOI:** 10.1177/10790632251350625

**Published:** 2025-06-27

**Authors:** Melissa O’Donaghy, Kelly M. Babchishin, Grace Culp, Rachael Zarbl, Alexis G. Hinkson

**Affiliations:** 1 6339 Carleton University, Ottawa, ON, Canada

**Keywords:** pedophilia, risk assessment, SSPI, SSPI-2, sexual recidivism

## Abstract

This study examined the convergent, divergent, predictive, and incremental validity of the Revised Screening Scale for Pedophilic Interests (SSPI-2) in a sample of 264 men adjudicated for sexual offenses against at least one child under the age of 15. We found evidence of construct validity as the SSPI-2 had small to medium correlations with phallometric testing (*r* = .31), recorded pedohebephilic diagnoses (*r* = .52), and attitudes tolerant of sexual offending against children (*r* = .23), in addition to small and non-significant correlations with the PCL-R (*r* = −.07), VRAG-R (*r* = −.09), BARR-2002R (*r* = −.06), and conduct disorder (*r* = −.07). As indicated by DeLong tests, the SSPI-2 was a better predictor of 5-year sexual (*Z* = −2.44) and non-contact sexual recidivism (*Z* = −2.46) than the SSPI. The SSPI-2 also added incremental predictive accuracy to risk tools such as the BARR-2002R, PCL-R, VRAG-R, and Static-99R. Overall, our findings suggest that the SSPI-2 is a valid measure of sexual interest in children and may be useful as a screening tool to help inform prioritization and management.

## Introduction

Pedophilia is an atypical sexual interest defined by persistent and ongoing sexual interest in prepubescent children (Seto, 2009). Given that pedophilic interest has been identified as a leading factor in the onset and maintenance of child sexual abuse (Hanson & Bussière, 1998; Hanson & Morton-Bourgon, 2004; Seto, 2018), it is an important factor to consider in the management of individuals with sexual offenses (Ramshaw et al., 2022). Several methods can be used to assess sexual interest in children, such as self-report (Banse et al., 2010), attentional or viewing tasks (Attard-Johnson et al., 2021; Babchishin et al., 2013b; Pedneault et al., 2021; Schmidt et al., 2017), and phallometric testing (i.e., measuring changes in penile erection in response to various stimuli to infer sexual arousal; McPhail et al., 2019). Not all measures are created equal. Some methods are more time-consuming (e.g., phallometric testing, self-report measures), invasive (e.g., phallometric testing), costly (e.g., phallometric testing), and may require direct contact with the individual (e.g., eye tracking measures). Sometimes direct contact with the client is not possible, or the client may refuse to participate. File-based measures of sexual interest in children offer a fast, valid, and cost-efficient method of assessing sexual interest in children. These file-based measures can be used with either archival or file-coded information, allowing for the retrospective coding of sexual interest in children.

The Screening Scale for Pedophilic Interests (SSPI) is a file-based measure designed to assess pedophilic interest based on offense characteristics for men with victims under the age of 14 (Seto & Lalumière, 2001). It is composed of four items: any boy victim(s), more than one victim, any prepubescent victim(s), and any extrafamilial victim(s). The Revised Screening Scale for Pedophilic Interests (SSPI-2) was developed within a sample of men with child victims under the age of 15 to incorporate an additional item: child pornography offenses (Seto et al., 2017b) based on the evidence that men charged with child pornography offenses had stronger penile responses to children than men without child pornography offenses (Seto et al., 2006; Seto, 2013). During this revision, Seto et al. (2017b) also found that the greater weight on the boy victim item (a score of two instead of one like the other items) in the SSPI was not necessary, and that reducing this weight yielded better concordance between the SSPI-2 and penile plethysmography. Given its recent development, only a few studies explored the validity of the SSPI-2 (e.g., Faitakis et al., 2023; Gouveia et al., 2024; Lalumière et al., 2024; Renaud, 2019; Seto et al., 2017a, 2017b; Sielaff et al., 2024; Stephens et al., 2019).

### Assessing Validity

Validating a measure can be accomplished by assessing the tool’s construct validity (i.e., a tool’s ability to measure what it claims to be measuring), which can be supported by convergent, divergent, predictive, and incremental validity (Campbell & Fiske, 1959). Convergent validity involves demonstrating that similar measures of the same constructs have strongly related scores (Gravetter & Forzano, 2018). Over the years, multiple studies have assessed the convergent validity of the SSPI and the SSPI-2 with related measures. As expected, the SSPI-2 is highly correlated with the SSPI (*r* = .96; Seto et al., 2017a). The SSPI-2 is also positively and significantly associated with measures of atypical sexual interest, such as a phallometric index of sexual arousal to children (*r* = .25; Seto et al., 2017b), self-reported pedophilic interests (*r* = .48; Seto et al., 2017b), a pedophilic diagnosis (*r* = .33; Sielaff et al., 2024), and the sexual deviance item of the Sexual Violence Risk-20 (*r* = .31; Gouveia et al., 2024). A meta-analysis conducted by Schmidt et al. (2017) found a significantly small, positive correlation between the SSPI and viewing time measures (*r* = .21). Additionally, SSPI total scores show a moderate correlation with the parole officer-scored deviant sexual interests item from the STABLE-2000 (*r *= .27) and STABLE-2007 (*r *= .55; Helmus et al., 2015). There is also a strong correlation between the SSPI and the Static-2002R’s *Deviant Sexual Interests* subscale (*r* = .70; Helmus et al., 2015).

Divergent validity is evidenced by small to null correlations between two or more measures that assess different constructs (Gravetter & Forzano, 2018). For instance, Lalumière et al. (2024) found minimal associations between the SSPI-2 and general criminal propensity measures, such as the Static-99R index violence (*r* = −.03) and prior non-sexual violence (*r* = −.002). Helmus et al. (2015) found negligible associations between SSPI scores and risk scale items that capture antisociality or general criminality, such as lack of cooperation with supervision (*r* = .07), impulsive acts (*r* = −.01), and break and enter convictions (*r* = −.02). Similarly, Seto et al. (2017a) found that neither the SSPI nor the SSPI-2 were significantly correlated with non-compliance with supervision (SSPI *r*_*pb*_ = −.009; SSPI-2 *r*_*pb*_ = −.012), general self-regulation problems (SSPI *r*_*pb*_ = .008; SSPI-2 *r*_*pb*_ = .005), and antisocial orientation (SSPI *r*_*pb*_ = −.010; SSPI-2 *r*_*pb*_ = .002). Additionally, both the SSPI and SSPI-2 have negligible associations with measures of psychopathy, such as the Psychopathy Checklist-Revised (PCL-R; *r*_SSPI_ = .10; Eher et al., 2015) and the Self-Report Psychopathy-Short Form (*r*_SSPI-2_ = −.18; Gouveia et al., 2024). Similarly negligible associations with measures of general criminality were found in Lalumière et al. (2024), leading the authors to conclude that the SSPI-2 can be best conceptualized as a measure of sexual interest in children rather than a propensity to commit sexual offenses.

Evidence for the validity of the measure can also be indicated by predictive validity, which refers to how well the measure of interest – the SSPI-2 – can predict a future relevant outcome, such as sexual reoffending. Overall, the SSPI-2 provides a small to moderate prediction of sexual reoffending (AUCs ranging from .43 to .71, *Mdn* = .62; see Table 1). This small to moderate relationship is not surprising, as more accurate predictions of sexual reoffending require both general and sexual criminality measures (Brouillette-Alarie et al., 2018, 2023; Hanson & Morton-Bourgon, 2004, 2005) and diagnostic tools such as the SSPI-2 are not risk assessment instruments, even though they may have predictive relevance.

**Table 1.** Predictive Validity of the SSPI and SSPI-2 in Previous Studies.

Study	Sample description	Follow-up period	*N*	Sexual recidivism		Violent recidivism		Any recidivism	
				AUC	[95% CI]	AUC	[95% CI]	AUC	[95% CI]
Canales et al. (2009): SSPI	Men with any child victim (≤14) in Canada	6.9 years	79	.47_charged._ 48_reconvicted_	[.33, .61] [.33, .62]	—	—	—	—
Eher et al. (2015): SSPI	Men from the Austrian prison system with at least one contact offense against a child (≤14)	6.3 years	189	**.71**	**[.57, ** **.86]**	—	—	—	—
Faitakis et al. (2023): SSPI-2	Men who were assessed at a sexual behavior clinic in Canada with one child victim (<15)	10 years	626	**.54**		.49		—	—
Helmus et al. (2015): SSPI	Men with sexual offense histories involving children (<14) and who were beginning a period of community supervision in Canada	7.5 years	365	**.62** .**64**_breaches_	**[.52, .72]** **[.56, .73]**	—	—	—	—
Moulden et al. (2009): SSPI	Men convicted of contact sexual offense against an unrelated, male or female child (<16) in Canada	20 years (fixed)	206	.47	—	.48	—	.47	—
McPhail et al. (2021): SSPI	Men convicted of sexual offenses against children (<14) in Canada	12.2 years	50	.63	[.47, .79]	—	—	—	—
Seto et al. (2004): SSPI	N_1_ – men convicted of at least one sexual offense against a child (<14) in Canada	5 years	113	.62	—	**.67**	—	—	—
	N_2_ – men who committed an offense involving sexual contact with a child (<14) in Canada and at least 5 years younger than the participant	5.3 years	145	**.69**	—	**.62**	—	—	—
Seto et al. (2017a): SSPI & SSPI-2	Men with child victims, deemed not in need of civil management in New York State (<14)	5 years (fixed)	856	**.60**_SSPI_ **.62**_SSPI-2_	**[.51, .69]** **[.53, .71]**	.51 _SSPI_ .53 _SSPI-2_	[.42, .59] [.44, .61]	.45 _SSPI_ .45 _SSPI-2_	[.41, .49] [.41, .49]
Shaal (2014): SSPI	Indiana-based sample of men with at least one sexual offense against a child (<14)	8.1 years	122	.43	[.21, .65]	—	—	—	—
Sielaff et al. (2024): SSPI-2	Men from the Austrian prison system with at least one contact sexual offense against a child (<15)	10.4 years	438	**.67**	**[.58, .76]**	—	—	—	—

*Note.* Bolded values are significant at the *p* < .05 level. AUCs were estimated based on the effect sizes reported in the articles. Follow-up periods are averages unless otherwise noted.

Psychosexual evaluations are complex and time-consuming. The main objective of many assessments is to determine risk management strategies, including intervention targets (Bonta & Andrews, 2024). Incremental validity refers to whether a measure of interest adds unique information above and beyond what is already being captured by another measure and can speak to construct validity (Horst, 1941). Two measures assessing similar constructs are expected to yield only marginal incremental improvements. In contrast, when two measures assess different constructs, they are likely to demonstrate meaningful incremental improvements. The extent to which the SSPI-2 improves recidivism prediction beyond available risk tools could also be a consideration for incorporating the SSPI-2 into the evaluator’s test battery. Similar to the literature on the predictive validity of the SSPI-2, there is limited research on the incremental validity of the SSPI-2 to other risk tools. Seto et al. (2004) found that the SSPI demonstrated incremental validity to the PCL-R in the prediction of sexual recidivism in a Canadian sample of 145 men adjudicated for sexual offenses against children. The SSPI, however, did not add to the predictive validity of the Static-99R or the Static-2002R, but did add to the predictive accuracy of the STABLE-2007 in a Canadian sample of 303 men adjudicated for sexual offenses against children (Helmus et al., 2015). Similarly, the SSPI-2 did not add incremental validity to the Static-99 in the prediction of sexual recidivism in an Austrian sample of 438 men convicted of sexual offenses against children (Sielaff et al., 2024). These results indicate that the predictive value of the SSPI/2 may already be adequately captured in the Static-99/R and Static-2002R.

### Current Study

The purpose of the current study is to provide an independent validation of the SSPI-2 by (1) examining its association with measures of similar constructs (convergent validity) and dissimilar constructs (divergent validity), (2) examining the differences between the SSPI and the SSPI-2 on predictive accuracy, and (3) assessing whether the SSPI-2 adds incremental validity to risk tools. Based on previous literature demonstrating positive associations between the SSPI-2 and measures of atypical sexual interests (e.g., Gouveia et al., 2024; Sielaff et al., 2024), it was hypothesized that the SSPI-2 will have convergent validity with measures that assess sexual interest in children and attitudes tolerant of sexual offending against children. It was also hypothesized that there will be divergent validity between the SSPI-2 and measures that assess antisociality and general criminality (e.g., PCL-R, VRAG-R, conduct disorder). Men adjudicated for Child Sexual Exploitation Material (CSEM) offenses are more likely to have sexual interests in children (Babchishin et al., 2018; Seto et al., 2006), which is a robust indicator of sexual reoffending (Hanson & Morton-Bourgon, 2004; Stephens et al., 2017). It was therefore anticipated that the SSPI-2 would be a better predictor of sexual recidivism than the original SSPI due to the addition of the child pornography item. Finally, based on previous research examining the SSPI and its incremental validity with the Static-99/R and Static-2002R (e.g., Helmus et al., 2015; Sielaff et al., 2024) and the high correlation between the SSPI and SSPI-2 (e.g., *r* = .96; Seto et al., 2017a), it was hypothesized that the SSPI-2 will not add meaningful incremental validity to these scales. Incremental validity was expected for the SSPI-2 to the BARR-2002R, PCL-R, and VRAG-R, given that these tools measure general criminality. Sexual recidivism is generally best predicted from items assessing both sexual and general criminality (Brouillette-Alarie et al., 2018; Hanson & Morton-Bourgon, 2004, 2005).

## Method

### Participants

The current study is a reanalysis of Hanson and Harris (1998), which was a convenience sample of 409 men convicted of at least one contact sexual offense in Canada. The original data was collected as part of a study on dynamic risk factors (Hanson & Harris, 1998), with recidivism information updated in 2017 (Aelick et al., 2020). Participants came from all provincial regions of Correctional Services of Canada and were undergoing supervision from 1987 to 1997 (*Mdn* = 1996; Hanson & Harris, 1998). Following the inclusion criteria of the SSPI-2, all participants without any child victims under the age of 15 (*n* = 117) and without at least a minimum 5-year follow-up (*n* = 28) were excluded from the current study. Therefore, our sample included 264 men adjudicated for sexual offenses against at least one child under 15.

Participants in this sample presented a higher risk of sexual recidivism, with an average Static-99R score of 4.20 (Level IVa – *Above Average Risk* category; Helmus et al., 2021), placing them at the 80^th^ percentile of risk compared to the typical population of men adjudicated for sexual offenses in Canada (Hanson et al., 2012). This elevated risk is attributable to the original study (Hanson & Harris, 1998) oversampling recidivists through a case-control design that compared recidivists to non-recidivists on acute and dynamic factors, therefore increasing the risk level of this sample. Descriptive statistics for the sample are presented in Table 2. Ethics approval was not required in the current study given that it involved secondary data analysis of anonymized data.

**Table 2.** Sample Characteristics.

Demographic variables	*n/N* (%)	*M*	*SD*
Ethnicity			
White	227/264 (86%)	–	–
Indigenous	22/264 (8.3%)	–	–
Black	7/264 (2.7%)	–	–
Asian	1/264 (0.4%)	–	–
Other^a^	7/264 (2.7%)	–	–
Marital status			
Married/common-Law	67/262 (25.6%)	–	–
Never married	101/262 (38.5%)	–	–
Separated/divorced	92/ 262 (35.1%)	–	–
Widowed	2/262 (0.8%)	–	–
Employment status at index			
Employed	80/259 (30.9%)	–	–
Unemployed	130/259 (50.2%)	–	–
Employed part-time	44/259 (17%)	–	–
Prior offenses			
# Prior offenses (sexual)	264	2.76	4.32
# Prior offenses (non-sexual violent)	264	0.95	2.01
# Prior offenses (other)	264	6.92	14.50
Age at index offense (years)	264	35.36	11.08
Age at release (years)	264	40.18	11.70
SSPI-2 total score	264	3.06	1.04
Static-99R total score	264	4.20	2.48
Five-year recidivism rate			
Any sexual	47/264 (17.8%)	–	–
Any contact sexual	29/264 (11.0%)	–	–
Any non-contact sexual	5/264 (1.9%)	–	–
Any violent (including contact sexual)	58/264 (22.0%)	–	–
Any crime^b^	145/260 (55.8%)	–	–
Ten-year recidivism rate			
Any sexual	74/256 (28.9%)	–	–
Any contact sexual	58/256 (22.7%)	–	–
Any non-contact sexual	5/256 (2.0%)	–	–
Any violent (including contact sexual)	92/256 (35.9%)	–	–
Any crime^b^	141/252 (56.0%)	–	–
Twenty-year recidivism rate			
Any sexual	62/178 (34.8%)	–	–
Any contact sexual	51/178 (28.7%)	–	–
Any non-contact sexual	8/178 (4.5%)	–	–
Any violent (including contact sexual)	69/178 (38.8%)	–	–
Any crime^b^	93/174 (53.4%)	–	–

*Note. N* = 264. The total number of recidivists in the “any contact sexual” and “any non-contact sexual” categories does not correspond to the number of recidivists in the “any sexual” category because the specific nature of the offenses (i.e., whether they were contact or non-contact) was not always available.

^a^Reflects participants identifying as Hispanic, Métis, Fijian, Moroccan, Tamil and El Salvadorean.

^b^Four participants were missing information on the status of any criminal recidivism.

### Measures

#### Sexual Domain Variables

##### Attitudes Tolerant of Sexual Offending Against Children

To compute this variable, four items derived from the Sexy Children subscale of the Hanson Sex Attitude Questionnaire (Hanson et al., 1994) were summed from the dataset: (1) child does not resist sexual touching – feels okay about it, (2) children are so willing to have sex – it is difficult to stay away, (3) some children are mature enough to enjoy sex with adults, and (4) some children like to sexually tease me. Community supervision officers evaluated these items based on interview data, scoring each item as follows: zero (no indication of attitudes tolerant of sexual offending against children), 1 (possible indication), or 2 (definite indication). Scores ranged from zero to 8, where higher values were indicative of attitudes that condone the sexual victimization of children (α = 0.89 in the current study).

##### Pedohebephilic Diagnosis

Pedohebephilia is defined as the sexual attraction to prepubescent and pubescent children (Seto, 2018). This item was coded dichotomously based on whether participants disclosed any pedohebephilic interests at the time of their sexual offense, or if there were any diagnoses for pedophilia/hebephilia in their criminal file. Based on practices at the time, it is likely that psychologists performed these assessments as part of pre-sentence or release reports, applying criteria from the DSM-III, DSM-IIIR, or DSM-IV.

##### Phallometric Testing

Penile plethysmography (PPG) is a physiological measure of penile sexual arousal, assessed through changes in penile volume or circumference (Seto et al., 2017b). In this study, phallometric testing data were drawn from file information, and PPG scores were calculated using the maximum dichotomized phallometric testing score of participants’ sexual preferences for girls, boys, and any children. PPG scores represent a difference score, comparing arousal levels to child versus adult stimuli. A positive Z-score signifies greater arousal to child stimuli compared to adult stimuli. In the dataset, PPG Z-scores were dichotomized such that a score of zero indicates greater interest in adult stimuli, while a score of 1 reflects stronger interest in child stimuli. Since participants in the study were from different provinces across Canada and PPG laboratories utilized different methodologies, both volumetric and circumference-based testing were used.

##### SSPI/2

The SSPI/2 are file-based measures that assess an individual’s sexual attraction to prepubescents. These tools were designed for men (aged 18 and older) who have committed at least one contact or non-contact sexual offense against a child (below the age of 15; Seto & Lalumière, 2001). SSPI/2 scores range from zero to 5, where higher scores represent a greater sexual interest in prepubescent children. The SSPI and SSPI-2 were scored using information from the dataset, with no missing data.

#### Risk Tools and General Criminality Variables

##### Brief Assessment for Recidivism Risk (BARR-2002R)

The BARR-2002R (Babchishin et al., 2012a, 2013a; see https://saarna.org) is an actuarial risk assessment tool designed to predict general and violent (including sexual) recidivism for adult males who have committed at least one sexual offense. The scale consists of the *General Criminality* subscale of the Static-2002R and *Age at Release*, where scores range between −2 and 8. For the current study, the BARR-2002R was scored based on the variables in the dataset, with no missing information. The BARR-2002R has been found to predict general and violent recidivism among men with sexual offense histories in line with more involved measures such as the Level of Service/Case Management Inventory and the Statistical Information on Recidivism scale (Babchishin et al., 2013a; Blais et al., 2022). In a study by Jung et al. (2018) of 342 men adjudicated for sexual offenses, large effect sizes for predictions of general and violent recidivism were observed (AUCs = .72 and .74, respectively), and a moderate effect size was observed for sexual recidivism (AUC = .66).

##### Conduct Disorder

Conduct disorder in childhood describes an individual’s history of persistent rule violations and antisocial tendencies. The conduct disorder scale used in this study consisted of 15 dichotomously scored items (e.g., has been physically cruel to animals) derived from Section A of the Conduct Disorder criteria in the DSM-IV, each scored on a two-point scale (0 = no evidence, 1 = evidence). This scale was completed by both the supervision officers and participants. Scores on the conduct disorder scale ranged from zero to 15, where higher scores indicate greater levels of antisociality (α = 0.78 in the current study).

##### Static-99R

The Static-99R (Helmus et al., 2012; see https://saarna.org) is an actuarial risk assessment tool designed to predict sexual recidivism among adult males (18+) with sexual offense histories. This tool consists of 10 items targeting factors pertaining to criminal history (e.g., prior sexual offenses), victim characteristics (e.g., any stranger victims), and relationship history (e.g., ever lived with a lover for at least two years). Scores on the Static-99R range from −3 to 12, with scores ranging from −3 to −2 representing a *very low risk* for sexual reoffending; scores of −1 to zero representing a *below average risk* for sexual reoffending; 1 to 3 representing an *average risk* for sexual reoffending; 4 to 5 representing the *above average risk* category; and scores of 6 or more capturing the *well above average risk* category. The Static-99R was scored based on the variables in the dataset, with two participants missing data for the ever lived with a partner item. This tool has consistently demonstrated strong interrater reliability in multiple studies (e.g., ICC = .91 in Hanson et al., 2015; ICC = .98 in Rettenberger et al., 2010; and ICC = .92 in Storey et al., 2012) and has been shown to have moderate predictive accuracy (e.g., random-effects AUC = .69, 95% CI = [.67, .72], *k*[studies] = 56, *N* = 71,515; Helmus et al., 2022).

##### Static-2002R

The Static-2002R (Helmus et al., 2012; see https://saarna.org) is an actuarial risk assessment tool intended for use among men with sexual offense histories. This tool consists of 14 items grouped into five subscales: Age at Release, Persistence of Sexual Offending, Sexual Deviance, Relationship to Victims, and General Criminality. Scores range from −2 to 13 and place individuals in one of five standardized risk categories. In the current study, the Static-2002R was scored based on variables in the dataset, with no missing data. The Static-2002R has been shown to predict sexual, violent, and any recidivism on par with the Static-99R (e.g., Babchishin et al., 2012b; Reeves et al., 2018).

##### Psychopathy Checklist-Revised (PCL-R)

The PCL-R (Hare, 2003) is a clinical assessment tool used to assess the two central factors of psychopathy, each with two facets. Factor 1 is composed of the interpersonal (e.g., superficial charm) and affective facets (e.g., lack of remorse), and Factor 2 captures the erratic lifestyle (e.g., impulsivity) and antisocial behavior (e.g., delinquency) components. In the current study, PCL-R total scores were transcribed from participants’ criminal files, with total scores available for 223 participants. Total scores range from zero to 40, with scores of 30 or higher meeting the threshold for psychopathy (Hare & Neumann, 2009). The PCL-R has demonstrated good interrater reliability (weighted ICC = .87, 95% CI [.84, .90], *k* = 4; Olver et al., 2020). The scale has also been identified as a strong predictor of violent (e.g., weighted *d* = .57, *k* = 9; DeMatteo & Olver, 2021), and general (any) recidivism (e.g., weighted *d* = .55, *k* = 7; DeMatteo & Olver, 2021), but is a weaker predictor of sexual recidivism (e.g., weighted *d =* .40, *k* = 4; DeMatteo & Olver, 2021).

##### Violence Risk Appraisal Guide-Revised (VRAG-R)

The VRAG-R is an actuarial risk-assessment tool designed to estimate men’s risk of violent recidivism within 5 to 12 years of community release (Rice et al., 2013). Consisting of 12 static predictors, VRAG-R items are scored based on the direction and magnitude of the item’s relationship with violent recidivism and are summed to obtain a total score that ranges between −34 and 44 (Quinsey, 2019). These values are then categorized and placed into nine bins, where higher VRAG-R scores are indicative of a greater risk of violent recidivism (Rice et al., 2013). This tool has demonstrated strong interrater reliability (e.g., ICC = .97; Olver & Sewall, 2018) and has been shown to have moderate predictive accuracy for both violent (AUC = .66) and general (any) recidivism (AUC = .66; Glover et al., 2017). VRAG total scores and items were coded directly from participants’ criminal files and entered into the dataset. VRAG-R scores were then computed via syntax from the dataset, with no missing items. See Table 1S of the Online Supplement for the descriptive statistics of the measures used in this study.

#### Recidivism

The current study examined five types of recidivism: (a) any recidivism, (b) non-contact sexual recidivism, (c) contact sexual recidivism, (d) any sexual recidivism, and (e) any violent (including contact sexual) recidivism. Recidivism was defined as any charges or convictions for new crimes. The any recidivism category includes sexual reoffending, violent reoffending, non-violent and non-sexual reoffending, as well as breaches in parole/probation conditions. The any sexual recidivism category includes both contact (e.g., sexual assault) and non-contact reoffending (e.g., exhibitionism). The current study analyzed recidivism over a fixed 5-year, 10-year, and 20-year period (see Table 2). Recidivism information was collected from the official records of the Canadian Police Information Center (CPIC) in 2017 (Aelick et al., 2020).

### Data Analyses

The authors take responsibility for the integrity of the data, the accuracy of the data analyses, and have made every effort to avoid inflating statistically significant results. We report how we determined our sample size, all data exclusions, all manipulations, and all measures in the study.

#### Polychoric and Polyserial Correlations

Polychoric correlations were computed to analyze correlations between non-continuous variables (Babchishin & Helmus, 2016; Flora & Curran, 2004; Holgado-Tello et al., 2010). Polyserial correlations were used to correlate ordinal (e.g., SSPI-2) with continuous variables (e.g., PCL-R). These correlation coefficients, rather than Pearson’s *r*, were used because ordinal scores (such as the SSPI-2’s total score) possess categorical restrictions, which tend to reduce data variability and artificially attenuate coefficients (Babchishin & Helmus, 2016). Polychoric and polyserial correlations, however, are less sensitive to the base rates of dichotomous data and ordinal data than Pearson’s *r* or point-biserial correlations. Polychoric and polyserial correlations that have values of .10, .30, and .50 correspond to small, moderate, and large effects, respectively (Cohen, 1992). The 95% confidence intervals (CI) were calculated using the following formula after Fischer’s transformation: CI = r ± (1.96 * *standard error*). A correlation is statistically significant at *p* < .05 if its 95% confidence interval does not include zero.

#### Expected Recidivism Rates

Expected recidivism rates were computed for cells with at least 10 participants from the logistic regression. Expected recidivism rates can be computed as B0 + *score* * B1. Logits were transformed into probabilities (p) to provide interpretable numbers, where p = 1 / (1 + e^−LOGIT^).

#### Predictive accuracy (i.e., discrimination)

A series of AUCs were conducted to index the predictive accuracy (i.e., discrimination) of the SSPI compared to the SSPI-2 using SPSS Version 28 (IBM SPSS, 2021). Rice and Harris (2005) describe the thresholds for small (AUC = .56), moderate (AUC = .64) and large (AUC = .71) effects when studying risk tools. AUCs are statistically significant if their 95% confidence intervals do not include .50. The DeLong test was used to determine whether the difference between two AUCs is statistically significant at *p* < .05 (DeLong et al., 1988). AUC values differ significantly from each other when the 95% confidence interval of the difference score does not include zero, which is also indexed by a significant Z-score. We also computed AUC values to index the predictive accuracy of the SSPI/2 in the prediction of pedophilic interests.

#### Incremental Analyses

Analyses of incremental validity used logistic regression to determine if a measure adds incrementally to another in predictions of recidivism (Helmus & Babchishin, 2017). In the context of this study, the odds ratio represents the increase or decrease in the odds of sexual recidivism, after controlling for the other measure. In the current study, we examined whether the SSPI-2 added incrementally to the SSPI, a series of risk tools (i.e., the BARR-2002R, Static-99R, Static-2002R, VRAG-R), and the PCL-R, in the prediction of 5-year sexual recidivism.

## Results

### Construct Validity

There was a strong, positive association between the SSPI-2 and the SSPI (*r* = .99). The SSPI-2 had a moderately positive relationship with other measures of sexual interest in children, such as a pedohebephilic diagnosis (*r* = .52), phallometric testing (*r* = .31), and attitudes tolerant of sexual offending against children (*r* = .23). The SSPI-2 had weak, non-significant correlations with measures of general criminality, such as the BARR-2002R (*r* = −.06), conduct disorder (*r* = −.07), PCL-R (*r* = −.07), and VRAG-R (*r* = −.09; see Table 3).

**Table 3.** Convergent and Divergent Validity of the SSPI-2.

Variable	*n*	*r*	[95% CI]
Convergent validity			
Attitudes tolerant of sexual offending against children	255	**.23**	**[0.11, 0.34]**
Pedohebephilic diagnosis	264	**.52**	**[0.43, 0.60]**
Phallometric testing	90	**.31**	**[0.11, 0.49]**
SSPI	264	**.996**	**[0.995, 0.997]**
Divergent validity			
BARR-2002R	264	−.06	[−0.18, 0.06]
Conduct disorder	264	−.07	[−0.19, 0.05]
PCL-R	223	−.07	[−0.20, 0.06]
VRAG-R	264	−.09	[−0.21, 0.03]

*Note.* Polychoric and polyserial correlation coefficients presented. Bolded correlations were statistically significant at *p* < .05; confidence intervals were calculated after Fisher’s transformation. SSPI-2 = Revised Screening Scale for Pedophilic Interests; SSPI = Screening Scale for Pedophilic Interests; BARR-2002R = Brief Assessment for Recidivism Risk; PCL-R = Psychopathy Checklist-Revised; VRAG-R = Violence Risk Appraisal Guide-Revised.

As SSPI and SSPI-2 scores increased, the prevalence of pedohebephilic diagnoses and PPG-assessed pedophilia also increased (see Table 4). AUC values were small to moderate when using the SSPI/2 to predict pedophilic interests.

**Table 4.** Prevalence of PPG, Pedohebephilic Diagnoses, and Any Indicator of Pedophilia per SSPI and SSPI-2 Scores.

	PPG-assessed pedophilia	Pedohebephilic diagnoses	Any indicator of pedophilia^a^
	*n/N* (%)	*n/N* (%)	*n/N* (%)
SSPI			
0	0/1 (0.0%)	1/3 (33.3%)	1/3 (33.3%)
1	4/10 (40.0%)	2/25 (8.0%)	6/25 (24.0%)
2	7/7 (100.0%)	13/31 (41.9%)	15/31 (48.4%)
3	20/21 (95.2%)	42/74 (56.8%)	44/74 (59.5%)
4	7/8 (87.5%)	18/26 (69.2%)	18/26 (69.2%)
5	35/43 (81.3%)	82/105 (78.1%)	83/105 (79.0%)
Total	73/90 (81.1%)	158/264 (59.8%)	167/264 (63.3%)
SSPI-2			
0	0/1 (0.0%)	1/3 (33.3%)	1/3 (33.3%)
1	4/10 (40.0%)	2/25 (8.0%)	6/25 (24.0%)
2	10/10 (100.0%)	19/37 (51.4%)	20/37 (54.1%)
3	24/26 (92.3%)	53/93 (57.0%)	56/93 (60.2%)
4	32/40 (80.0%)	78/101 (77.2%)	79/101 (78.2%)
5	3/3 (100.0%)	5/5 (100.0%)	5/5 (100.0%)
Total	73/90 (81.1%)	158/264 (59.8%)	167/264 (63.3%)

*Note.* PPG = penile plethysmography.

^a^Any indicator of pedophilia was computed by using the maximum score between PPG-assessed pedophilia and pedohebephilic diagnoses. AUC_SSPI predicting PPG-assessed pedophilia_ = .59 [.41, .76], AUC_SSPI-2 predicting PPG-assessed pedophilia_ = .60 [.43, .77], AUC_SSPI predicting pedohebephilic diagnoses_ = .72 [.66, .79], AUC_SSPI-2 predicting pedohebephilic diagnoses_ = .71 [.65, .77], AUC_SSPI predicting any indicator of pedophilia_ = .69 [.63, .76], AUC_SSPI-2 predicting any indicator of pedophilia_ = .69 [.62, .75].

### Predictive Accuracy (Discrimination)

After a fixed 5-year follow-up period, the SSPI-2 predicted any sexual recidivism (AUC_SSPI_ = .57, AUC_SSPI-2_ = .60; DeLong *Z* = −2.44, *p* = .015) and non-contact sexual recidivism (AUC_SSPI_ = .63, AUC_SSPI-2_ = .73; DeLong *Z* = −2.46, *p* = .014) better than the SSPI. There were no statistically significant differences between the SSPI and SSPI-2 at five years for violent, contact sexual, or general reoffending (AUC difference values ranged between −.011 and −.099, *Mdn* = −.016; see Table 5).

**Table 5.** Predictive Validity of the SSPI and SSPI-2 for Fixed 5-Year, 10-Year, and 20-Year Any Sexual Recidivism.

Recidivism type	AUC [95% confidence interval]	Difference [95% CI]	*Z*
Sex any 5-year		**−.025 ** **[−.04, ** **−.01]**	**−2.44**
SSPI	.57 [.49, .66]		
SSPI-2	**.60 ** **[.52, .68]**		
Sex any 10-year		−.010 [−.03, .008]	−1.08
SSPI	**.60 ** **[.53, .67]**		
SSPI-2	**.61 ** **[.54, .68]**		
Sex any 20-year		−.007 [−.03, .02]	−0.59
SSPI	.54 [.46, .62]		
SSPI-2	.55 [.46, .63]		
Sex contact 5-year		−.016 [−.03, .002]	−1.73
SSPI	.58 [.47, .69]		
SSPI-2	.60 [.49, .70]		
Sex contact 10-year		.0001 [−.019, .019]	−.009
SSPI	**.59 [.51, .67]**		
SSPI-2	**.59 [.51, .67]**		
Sex contact 20-year		−.005 [−.03, .02]	−0.38
SSPI	.54 [.44, .63]		
SSPI-2	.54 [.45, .63]		
Sex non-contact 5-year		**−.099 [**−**.18,** −**.02]**	**−2.46**
SSPI	.63 [.42, .84]		
SSPI-2	.73 [.48, .98]		
Sex non-contact 10-year		−.084 [−.17, .004]	−1.88
SSPI	**.72 [.54, .89]**		
SSPI-2	**.80 [.60, 1.01]**		
Sex non-contact 20-year		**−.058 [**−**.10,** −**.01]**	**−2.51**
SSPI	.59 [.42, .77]		
SSPI-2	.65 [.49, .82]		
Violent 5-year		−.011 [−.03, .004]	−1.42
SSPI	.51 [.43, .60]		
SSPI-2	.52 [.44, .60]		
Violent 10-year		−.013 [−.03, .005]	−1.40
SSPI	.51 [.44, .58]		
SSPI-2	.53 [.46, .60]		
Violent 20-year		−.011 [−.03, .01]	−0.92
SSPI	.49 [.41, .58]		
SSPI-2	.50 [.42, .59]		
Any crime 5-year		−.012 [−.03, .006]	−1.29
SSPI	.48 [.41, .55]		
SSPI-2	.49 [.42, .56]		
Any crime 10-year		−.010 [−.028, .008]	−1.07
SSPI	.48 [.41, .55]		
SSPI-2	.49 [.42, .56]		
Any crime 20-year		−.014 [−.04, .01]	−1.14
SSPI	.45 [.37, .54]		
SSPI-2	.47 [.39, .55]		

*Note.* Bolded values are significant at the *p* < .05 level. Negative difference scores indicate that the SSPI-2 is a better predictor than the SSPI*. N* = 264 for all 5-Year recidivism types with the exception for Any Crime (*N* = 260), *N* = 256 for all 10-Year recidivism types with the exception for Any Crime (*N* = 252), and *N* = 178 for all 20-Year recidivism types with the exception for Any Crime (*N* = 174).

The differences in the predictive accuracy of the SSPI and SSPI-2 were less marked after 10 years (AUC difference values ranged between −.084 and .0001, *Mdn* = −.010) and 20 years (AUC difference values ranged between −.005 and −.058, *Mdn* = −.011). Yet, there was a notable difference in predictive accuracy for non-contact sexual recidivism at the 20-year mark, where the SSPI-2 demonstrated significantly better predictive ability compared to the SSPI (AUC_SSPI_ = .59, AUC_SSPI-2_ = .65; DeLong *Z* = −2.51, *p* = .012).

As SSPI and SSPI-2 scores increased, both observed and expected sexual recidivism percentages tended to rise over time. This pattern is consistent across the 5-year, 10-year, and 20-year follow-up periods (see Table 2S of the Online Supplement).

### Incremental Validity

The SSPI-2 added incrementally to the BARR-2002R (OR = 1.58, Wald = 6.23, *p* = .013), where after controlling for the BARR-2002R, each additional point on the SSPI-2 was associated with a 58% increase in the odds of any sexual recidivism. The SSPI-2 also added incremental validity to the PCL-R (OR = 1.51, Wald = 4.68, *p* = .031), VRAG-R (OR = 1.63, Wald = 6.96, *p* = .008), and Static-99R (OR = 1.43, Wald = 3.94, *p* = .047; see Table 6). However, the SSPI-2 did not add incremental validity to the Static-2002R. See Table 4S of the Online Supplement for incremental analyses for other recidivism outcomes (i.e., violent and any recidivism).

**Table 6.** Incremental Validity of the SSPI-2 with Risk Tools for Predictions of Any, Contact, and Non-Contact Sexual Recidivism.

	Any sexual recidivism (5-year)				
	*N*	Odds ratio	[95% CI]	Wald	*p*
BARR-2002R	264	**1.26**	**[1.06, 1.50]**	**6.71**	**.010**
SSPI-2		**1.58**	**[1.10, 2.26]**	**6.23**	**.013**
PCL-R	223	**1.06**	**[1.02, 1.11]**	**6.95**	**.008**
SSPI-2		**1.51**	**[1.04, 2.19]**	**4.68**	**.031**
Static-99R	264	1.13	[0.98, 1.30]	2.97	.085
SSPI-2		**1.43**	**[1.01, 2.04]**	**3.94**	**.047**
Static-2002R	264	**1.17**	**[1.02, 1.34]**	**5.01**	.**025**
SSPI-2		1.36	[0.94, 1.95]	2.69	.101
VRAG-R	264	**1.04**	**[1.02, 1.07]**	**11.07**	**<.001**
SSPI-2		**1.63**	**[1.14, 2.35]**	**6.96**	**.008**
	Contact sexual recidivism (5-year)				
	BARR-2002R	264	**1.30**	**[1.04, 1.61]**	**5.31**	.**021**
	SSPI-2		1.45	[0.95, 2.23]	2.92	.087
PCL–R	223	**1.08**	**[1.02, 1.15]**	**7.14**	.**008**
SSPI-2		1.40	[0.88, 2.20]	2.03	.154
Static-99R	264	**1.26**	**[1.06, 1.51]**	**6.62**	**.010**
SSPI-2		1.26	[0.83, 1.92]	1.15	.284
Static-2002R	264	**1.27**	**[1.06, 1.51]**	**6.73**	**.010**
SSPI-2		1.18	[0.77, 1.83]	0.57	.450
VRAG-R	264	**1.04**	**[1.01, 1.07]**	**7.73**	**.005**
SSPI-2		1.50	[0.97, 2.31]	3.32	.069
	Non-contact sexual recidivism (5-year)				
	BARR-2002R	264	1.76	[0.88, 3.50]	2.59	.108
	SSPI-2		**5.95**	**[1.15, 30.80]**	**4.51**	**.034**
PCL–R	223	1.11	[0.97, 1.27]	2.43	.119
SSPI-2		**4.88**	**[1.02, 23.28]**	**3.95**	**.047**
Static-99R	264	1.14	[0.75, 1.75]	0.37	.541
SSPI-2		4.71	[0.96, 23.17]	3.64	.056
Static-2002R	264	1.56	[0.92, 2.62]	2.74	.098
SSPI-2		3.80	[0.74, 19.37]	2.58	.109
VRAG-R	264	1.07	[0.99, 1.16]	2.87	.090
SSPI-2		**7.15**	**[1.14, 44.94]**	**4.40**	**.036**

*Note.* Bolded values reached *p* < .05. SSPI-2 = Revised Screening Scale for Pedophilic Interests; BARR-2002R = Brief Assessment for Recidivism Risk; PCL-R = Psychopathy Checklist-Revised; VRAG-R = Violence Risk Appraisal Guide-Revised.

## Discussion

Given that sexual interest in children is one of the best predictors of sexual recidivism (e.g., Hanson & Morton-Bourgon, 2004), accurate assessments of sexual interest in children are of critical importance to inform the management of individuals who have committed sexual offenses. File-based measures, such as the SSPI-2, offer a convenient and cost-efficient method of assessing sexual interest in children. This study provided an independent validation of the SSPI-2 within a sample of 264 men adjudicated for sexual offenses against at least one child under the age of 15. We found evidence of convergent validity; the SSPI-2 was associated with a pedohebephilic diagnosis, phallometric measures, and attitudes tolerant of sexual offending against children. The correlation between the SSPI-2 and the phallometric pedophilic index (*r* = .31) in our subsample of 103 men with PPG information was similar to Seto et al.'s (2017b; *r* = .25, *p* < .001, *N* = 948) and Lalumière et al.’s (2024, *r* = .28, 95% CI [.22, .30], *N* = 1953).

Consistent with previous research (e.g., Eher et al., 2015; Gouveia et al., 2024; Helmus et al., 2015; Lalumière et al., 2024; Seto et al., 2017a), we found that the SSPI-2 expressed divergent validity with measures of general criminality, such as the BARR-2002R, PCL-R, VRAG-R, and conduct disorder. These findings further support that the SSPI-2 was not designed to measure antisociality or general criminality, but rather pedophilic sexual interests. Our divergent validity findings align with Lalumière et al.’s (2024) conclusion that the SSPI-2 reflects pedophilic interests rather than a general behavioral tendency to offend against children. SSPI-2 scores are more closely associated with indicators of sexual interest in children (e.g., pedophilia and pedohebephilia indices) than with general criminality indicators (e.g., prior sentencing dates, history of non-violent sexual offenses).

In the current study, higher SSPI and SSPI-2 scores were associated with increased rates of pedohebephilic diagnoses and PPG-assessed pedophilic interests. This pattern is consistent with prior research demonstrating that higher SSPI-2 scores are associated with a greater prevalence of pedophilic (Renaud, 2019) and pedohebephilic diagnoses (Stephens et al., 2019). Additionally, SSPI-2 scores in the current study were moderately associated with both clinical diagnoses of pedohebephilia and PPG-assessed pedophilic interests. However, given the modest effect size when associating SSPI-2 scores with sexual interest indicators, we recommend that the SSPI-2 not be used as a standalone alternative to comprehensive assessments, but rather as a screening tool to help prioritize cases for further evaluation.

We found that the SSPI-2 added incrementally to measures of general criminality, such as the BARR-2002R, PCL-R, and VRAG-R, in predicting sexual recidivism. On the other hand, the SSPI-2 did not add any predictive validity to the Static-2002R, suggesting that the SSPI-2 might be measuring the same construct that is already being captured in the Static-2002R (i.e., sexual criminality). Contrary to Seto et al. (2017a), we found that after controlling for the SSPI-2, the Static-99R did not predict any sexual recidivism. We speculate that this differential effect could be due to a power issue, as our study had a comparatively smaller sample size (*N* = 264) compared to Seto et al.'s (2017a, *N* = 856). The differential effect could also be related to the nature of our sample, as the average Static-99R score of participants in our study was considerably higher (4.20, *Above Average Risk*) than Seto et al.'s (2017a) sample (1.94, *Average Risk*). Therefore, participants in the current study had a higher risk for sexual reoffending, with consistently higher scores across the sample. This restricted range may have reduced the statistical power to detect an effect.

We found that both the SSPI and SSPI-2 predicted sexual recidivism rates at 5- and 20-year follow-ups, and that as scores on these scales increased, expected sexual recidivism rates also increased. More specifically, we found that the SSPI-2 was a better predictor of any sexual recidivism than the SSPI, meaning that the addition of the child pornography item and reweighting of the any boy victim(s) item improved the tool’s prediction of sexual reoffending.

We believe that the addition of the child pornography item improved the SSPI-2’s prediction of non-contact sexual recidivism, based on the evidence that CSEM offending is an indicator of pedophilic interests (Seto et al., 2006), that individuals adjudicated for such offenses are likely to reoffend with a CSEM offense (Babchishin et al., 2023), and that those with pedophilic interests are at greater risk for CSEM offending (Dombert et al., 2016).

Differences between SSPI and SSPI-2 scores are influenced by two key factors: the addition of the CSEM item in the SSPI-2 and the revised scoring of the any boy victim(s) item. If boy victims are more common than CSEM offenses, the revised scoring for the boy victim(s) item may offset or even outweigh the effect of adding CSEM, resulting in smaller score differences between the two versions. Conversely, if men with boy victims are also more likely to have CSEM offenses, the changes may compound, leading to larger differences in scores. Given that CSEM offenses and male victims are established indicators of pedophilic interests (e.g., Seto et al., 2006; Seto & Lalumière, 2001), the two revisions of the scale are likely influencing scoring. Additionally, the increasing availability of digital content may contribute to a higher prevalence of CSEM offenses in more recent samples. If more men gain points for CSEM than lose points due to the adjusted boy victim scoring, average SSPI-2 scores may rise. Over time, this could narrow the difference between SSPI and SSPI-2 scores. Ultimately, the impact depends on how often CSEM offenses and boy victims co-occur, underscoring the need for further empirical research.

### Practical Implications

Effective correctional programs match the intensity of treatment to the individual’s risk level and target their criminogenic needs (Bonta & Andrews, 2024; Hanson et al., 2009). Many measures of sexual interest in children require substantial resources (e.g., cost and time) to score (Seto, 2018). Treatment programs and correctional agencies could benefit from using the SSPI-2 to expedite their evaluation processes by allowing them to assess sexual interest in children quickly and efficiently. The SSPI-2 could also be used as a screening measure for pedophilic diagnoses during the assessment stage, helping to identify individuals who would benefit from more detailed, time-consuming evaluations, such as phallometric measures. For example, a SSPI-2 score of 4 was associated with a pedohebephilic diagnosis rate of 77% and an 80% rate of PPG-assessed pedophilia. The SSPI-2 could be used as an additional tool in comprehensive psychological assessments for higher-stake evaluations, such as the Dangerous Offender designation in Canada or the Sexually Violent Predator determination in the United States. The SSPI-2 could serve as a reliable predictor of non-contact sexual recidivism, as evidenced by its strong predictive ability (5-year AUC = .73). However, the SSPI-2 alone would not be sufficient for higher-stakes evaluations. High-stake evaluations should include multiple measures, as multimethod assessments minimize validity limitations that are present across different types of measures and offer a more accurate assessment of the construct (Hopwood & Bornstein, 2014).

Researchers could also use the SSPI-2 to retrospectively score sexual interest in children should they not have a measure of sexual interest in children in their dataset. As demonstrated in the current study, SSPI-2 scores can be obtained efficiently from an archival dataset and have high correlations with other measures of sexual interest in children, such as phallometrically-assessed sexual arousal to children.

### Limitations and Future Directions

The SSPI-2 demonstrated predictive validity for any sexual recidivism and non-contact sexual recidivism. However, we did not code for CSEM reoffending in the current study. If so, we could have examined if the improvement in the prediction of non-contact sexual recidivism for the SSPI-2 (compared to the SSPI) was attributable to the SSPI-2’s ability to predict CSEM reoffending. Future research should examine whether the SSPI-2’s improved predictive validity is specifically due to the reweighting of the any boy victim(s) item or the addition of the child pornography item. Using a larger and more contemporaneous sample would ensure sufficient statistical power to determine which item had the greatest impact on the performance of the SSPI-2.

The current study relied on an older sample of individuals released on community supervision between 1987 and 1997. While this allows for long follow-up periods, it also means that our sample is different from more contemporary samples. CSEM offenses were relatively unknown by law enforcement and were underresearched in the late 90s and early 2000s (Babchishin et al., 2018; Seto, 2013). The nature of the offense during this period was also very different than today. Acquiring CSEM was expensive, the speed of exchange was slow, and CSEM offending was primarily a solitary crime due to the difficulty of contacting others (Beech et al., 2008; Westlake, 2020). With technological advances, perpetrators have new avenues to lure and groom their victims, and they can now easily connect with like-minded users globally, thereby increasing peer-to-peer sharing (Westlake, 2020). Between 2010 and 2017, reported CSEM offenses in Canada increased by 288% (Department of Justice Canada, 2019). Our sample therefore had fewer CSEM offenses compared to more contemporaneous samples. For example, 7.6% (*n* = 20) of participants in our sample had committed a CSEM offense, whereas in a cohort of men under supervision for sexual offenses against children (*N* = 1350) in British Columbia from 2008 to 2013, 23% (*n* = 308; Babchishin et al., 2023) had committed a CSEM offense. As such, we would anticipate a more contemporary sample to have more CSEM offense histories and, therefore, differences between the SSPI and SSPI-2 may be larger in a more recent sample. Indeed, the correlation between the SSPI-2 and SSPI in our sample (*r* = .99) is larger than in other studies with more contemporaneous samples (*r* = .96; Seto et al., 2017a). Despite this large correlation, we still found that the SSPI-2 outperformed the SSPI in the prediction of any sexual recidivism and non-contact sexual recidivism.

Finally, future research should investigate whether the SSPI-2 expresses convergent validity with other measures of sexual interest in children (e.g., viewing time), given that previous research has mostly examined its relationship with risk tools and/or phallometrically-assessed sexual arousal to children (e.g., Helmus et al., 2015; Seto et al., 2017a). Examining the SSPI-2’s relationship with more varied measures of sexual interests could further validate its ability to assess pedophilic interests.

### Concluding Remarks

The current study adds to the growing body of evidence supporting the SSPI-2 as a valid indicator of sexual interest in children, as well as a useful predictor of sexual reoffending, particularly for non-contact sexual offenses. We found evidence of small to moderate convergent validity with measures of sexual interest, such as phallometric testing. Through this independent validation, the SSPI-2 outperformed the SSPI in predicting any sexual and non-contact sexual recidivism, suggesting that the SSPI-2’s predictive ability is improved by the addition of the child pornography item and reweighting of the any boy victim(s) item. We also found that as SSPI and SSPI-2 scores increased, the prevalence of pedohebephilic diagnoses and PPG-assessed pedophilia also increased. Further, observed and expected sexual recidivism percentages tended to rise over time. This could have practical implications for the assessment of pedophilic interests, where the SSPI-2 can serve as an easy method to prioritize cases for treatment or to screen cases that would benefit from more resource-intensive assessments of sexual interest in children.

## Supplemental Material

Supplemental Material - An Efficient Measure of Sexual Interest in Children: The Revised Screening Scale for Pedophilic Interests (SSPI-2) Supplemental Material for An Efficient Measure of Sexual Interest in Children: The Revised Screening Scale for Pedophilic Interests (SSPI-2) by Melissa O’Donaghy, Kelly M. Babchishin, Grace Culp, Rachael Zarbl, and Alexis Hinkson in Sexual Abuse
