# Supplementary material for: An Efficient Measure of Sexual Interest in Children: The Revised Screening Scale for Pedophilic Interests (SSPI-2)
Source: Sex Abuse. 2025 Jun 27;37(8):950–75. doi: 10.1177/10790632251350625 (PMC12552764; doi:10.1177/10790632251350625)
Supplement: Supplemental Material - An Efficient Measure of Sexual Interest in Children: The Revised Screening Scale for Pedophilic Interests (SSPI-2) [file sj-pdf-1-sax-10.1177_10790632251350625.pdf]

Online Supplementary Material

An Efficient Measure of Sexual Interest in Children: The Revised Screening Scale for Pedophilic Interests (SSPI-2)

Melissa O'Donaghy, Kelly M. Babchishin, Grace Culp, Rachael Zarbl, & Alexis Hinkson

Sexual Abuse

Table of Contents

Table 1S. Descriptives Statistics of Study Measures. 2

Table 2S. Observed and Expected Sexual Recidivism Rate for Fixed 5-Years, 10-Years, and 20-Years per SSPI and SSPI-2 Scores 3

Table 3S. Logistic Regression Estimates to Compute Expected Sexual Recidivism Rates 4

Table 4S. Incremental Analyses for Other Recidivism Outcomes 5

Table 1S.

Descriptive Statistics of Study Measures

Measure	*N*	*n* (%)	*M (SD)*	Min.	Max.
Sexual Domain Variables					
Attitudes tolerant of sexual offending against children	255	–	4.34 (3.15)	0	8
Pedohebephilic diagnosis	264	158 (59.8%)	–	0	1
Phallometric Testing	90	73 (81.1%)	–	0	1
SSPI	264	–	3.55 (1.41)	0	5
SSPI-2	264	–	3.06 (1.04)	0	5
Risk Tools and General Criminality Variables					
BARR-2002R	264	–	3.60 (2.08)	– 2	7
Conduct disorder	264	–	4.12 (2.84)	1	12
Static-99R	264	–	4.20 (2.48)	– 3	10
Static-2002R	264	–	6.18 (2.62)	– 1	11
PCL-R	223	–	19.39 (8.28)	1	37
VRAG-R	264	–	8.66 (15.27)	– 28	41

Note. Percentages indicate the proportion of participants scoring one for dichotomous variables. Minimum and maximum values represent the range within the dataset. SSPI = Screening Scale for Pedophilic Interests; SSPI-2 = Revised Screening Scale for Pedophilic Interests; BARR-2002R = Brief Assessment for Recidivism Risk; PCL-R = Psychopathy Checklist-Revised; VRAG-R = Violence Risk Appraisal Guide-Revised.

Table 2S.

Observed and Expected Sexual Recidivism Rate for Fixed 5-Years, 10-Years, and 20-Years per SSPI and SSPI-2 Scores

Scores	5-Year Sexual Recidivism Rate *n/N* (%)		10-Year Sexual Recidivism Rate *n/N* (%)		20-Year Sexual Recidivism Rate *n/N* (%)	
SSPI	Observed	Expected	Observed	Expected	Observed	Expected
0	0/3 (0.0%)	-[a]	1/2 (50.0%)	-	1/1 (100.0%)	-
1	2/25 (80.0%)	11.0%	3/25 (12.0%)	16.7%	4/16 (25.0%)	29.2%
2	5/31 (16.1%)	13.2%	4/30 (13.3%)	20.7%	5/19 (26.3%)	31.3%
3	14/74 (18.9%)	15.8%	23/72 (31.9%)	25.4%	20/57 (35.1%)	33.5%
4	2/26 (7.7%)	18.8%	6/25 (24.0%)	30.7%	7/19 (36.8%)	35.8%
5	24/81 (29.6%)	22.2%	37/102 (36.3%)	36.6%	25/66 (37.8%)	38.1%
Total	47/264 (17.8%)		74/256 (28.9%)		62/178 (34.8%)	
SSPI-2						
0	0/3 (0.0%)	-	1/2 (50.0%)	-	1/1 (100.0%)	-
1	2/25 (8.0%)	7.9%	3/25 (12.0%)	14.0%	4/16 (25.0%)	27.1%
2	5/37 (13.5%)	11.6%	5/36 (13.9%)	19.9%	6/26 (23.1%)	30.7%
3	16/93 (17.2%)	16.6%	28/90 (31.1%)	27.5%	26/68 (38.2%)	34.6%
4	21/101 (20.1%)	23.3%	34/98 (34.7%)	36.7%	24/66 (36.4%)	38.7%
5	3/5 (60.0%)	-	3/5 (60.0%)	-	1/1 (100.0%)	-
Total	47/264 (17.8%)		74/256 (28.9%)		62/178 (34.8%)	

Note. Any sexual recidivism was defined as any charges or convictions for new crimes (including breaches) for both contact and non-contact sexual offences. Median score of the SSPI and SSPI-2 in this sample was 3. Table 4S provides B0 and B1 used to compute the expected recidivism rate.

[a]Expected recidivism rates were not computed in cells with $n < 10$.

Table 3S.

Logistic Regression Estimates to Compute Expected Sexual Recidivism Rates

Measure	Outcome	B0	B1
SSPI	5-year sexual recidivism	.208	-1.672
	10-year sexual recidivism	.264	-1.077
	20-year sexual recidivism	.100	-.684
SSPI-2	5-year sexual recidivism	.422	-1.613
	10-year sexual recidivism	.425	-.968
	20-year sexual recidivism	.176	-.638

Note. SSPI and SSPI-2 scores are centered on the median score of 3 for both scales.

Table 4S.

Incremental Analyses for Other Recidivism Outcomes

	Violent (Including Contact Sexual) Recidivism (5-Year)				
	N	Odds Ratio	95% CI	Wald	*p*
BARR-2002R	264	**1.60**	**[1.32, 1.95]**	**22.79**	**<.001**
SSPI-2		1.12	[0.83, 1.51]	0.56	.455
PCL-R	223	**1.10**	**[1.05, 1.15]**	**16.19**	**<.001**
SSPI-2		1.02	[0.74, 1.40]	0.02	.898
Static-99R	264	**1.30**	**[1.14, 1.49]**	**14.49**	**<.001**
SSPI-2		0.94	[0.70, 1.26]	0.15	.696
Static-2002R	264	**1.29**	**[1.13, 1.47]**	**13.84**	**<.001**
SSPI-2		0.88	[0.65, 1.19]	0.66	.418
VRAG-R	264	**1.06**	**[1.04, 1.09]**	**25.34**	**<.001**
SSPI-2		1.15	[0.85, 1.56]	0.83	.364
	Any Recidivism (5-Year)				
BARR-2002R	260	**1.81**	**[1.54, 2.13]**	**50.66**	**<.001**
SSPI-2		1.07	[0.82, 1.41]	0.27	.605
PCL-R	221	**1.10**	**[1.06, 1.15]**	**25.45**	**<.001**
SSPI-2		0.92	[0.70, 1.22]	0.31	.578
Static-99R	260	**1.58**	**[1.39, 1.80]**	**46.29**	**<.001**
SSPI-2		0.78	[0.59, 1.04]	2.97	.085
Static-2002R	260	**1.52**	**[1.34, 1.72]**	**43.02**	**<.001**
SSPI-2		**0.72**	**[0.54, 0.96]**	**5.20**	**.023**
VRAG-R	260	**1.08**	**[1.05, 1.10]**	**44.41**	**<.001**
SSPI-2		1.08	[0.83, 1.40]	0.32	.570

Note. Bolded values reached $p < .05$.
